# Evaluation of SARS-CoV-2-Neutralizing Nanobody Using Virus Receptor Binding Domain-Administered Model Mice

**DOI:** 10.34133/2022/9864089

**Published:** 2022-07-22

**Authors:** Song Liu, Guanghui Li, Lei Ding, Jin Ding, Qian Zhang, Dan Li, Xingguo Hou, Xiangxing Kong, Jing Zou, Shiming Zhang, Hongbin Han, Yakun Wan, Zhi Yang, Hua Zhu

**Affiliations:** ^1^Key Laboratory of Carcinogenesis and Translational Research (Ministry of Education/Beijing), NMPA Key Laboratory for Research and Evaluation of Radiopharmaceuticals, Department of Nuclear Medicine, Peking University Cancer Hospital & Institute, Beijing 100142, China; ^2^Institute of Biomedical Engineering, Peking University Shenzhen Graduate School, Shenzhen, Guangdong 518055, China; ^3^Shanghai Novamab Biopharmaceuticals Co., Ltd., Shanghai, China; ^4^Key Laboratory of Carcinogenesis and Translational Research (Ministry of Education/Beijing), Department of Anesthesiology, Peking University Cancer Hospital & Institute, Beijing 100142, China; ^5^Institute of Medical Technology, Peking University Health Science Center, Beijing 100191, China; ^6^Beijing Key Laboratory of Magnetic Resonance Imaging Devices and Technology, Peking University Third Hospital, Beijing 100191, China; ^7^Department of Radiology, Peking University Third Hospital, Peking University, Beijing 100191, China

## Abstract

Due to the rapid spread of coronavirus disease 2019 (COVID-19), there is an urgent requirement for the development of additional diagnostic tools for further analysis of the disease. The isolated nanobody Nb11-59 binds to the severe acute respiratory syndrome coronavirus 2 (SARS-CoV-2) receptor-binding domain (RBD) with high affinity to neutralize the virus and block the angiotensin-converting enzyme 2- (ACE2-) RBD interaction. Here, we introduce a novel nanobody-based radiotracer named ^68^Ga-Nb1159. The radiotracer retained high affinity for the RBD and showed reliable radiochemical characteristics both *in vitro* and *in vivo*. Preclinical positron emission tomography (PET) studies of ^68^Ga-Nb1159 in mice revealed its rapid clearance from circulation and robust uptake into the renal and urinary systems. Fortunately, ^68^Ga-Nb1159 could specifically reveal the distribution of the RBD in mice. This study also helped to evaluate the pharmacodynamic effects of the neutralizing nanobody. Moreover, ^68^Ga-Nb1159 may be a promising tool to explore the distribution of the RBD and improve the understanding of the virus. In particular, this study identified a novel molecular radioagent and established a reliable evaluation method for specifically investigating the RBD through noninvasive and visual PET technology.

## 1. Introduction

The worldwide spread of coronavirus disease 2019 (COVID-19), which is caused by severe acute respiratory syndrome coronavirus 2 (SARS-CoV-2), has resulted into a global epidemic, posed threats to human health, and caused economic crises. SARS-CoV-2 is an enveloped and positive-sense single-stranded RNA virus. The genome of SARS-CoV-2, similar to that of SARS-CoV, encodes four structural proteins: spike (S), envelope (E), membrane (M), and nucleocapsid (N) [1]. The ectodomain of the spike protein contains a receptor-binding subunit (S1) and a membrane fusion subunit (S2). In the process of virus entry into host cells, the S1 subunit binds to a receptor on the surface of the host cells, and the S2 subunit is responsible for the fusion of the viral and cellular membranes. Receptor binding and membrane fusion between the virus and host cells play important roles in the viral infection cycle [2]. Angiotensin-converting enzyme 2 (ACE2) has been shown to be a functional receptor for SARS-CoV-2, with an effective interaction between the receptor-binding domain (RBD) of the spike protein and human ACE2 [3, 4]. Membrane fusion of the virus and the host cell is activated after binding, and viral RNA is subsequently released into the cytoplasm, establishing infection. Some transmembrane proteinases participate in this process [5]. Furthermore, a crystal structure of the SARS-CoV-2 RBD bound to ACE2 has been described, revealing the mechanism of underlying infection and facilitating the search for effective therapies [6, 7]. Mutations of the RBD can potentially provide more efficient contacts with human ACE2 and increase the infectivity of SARS-CoV-2 [3, 8, 9]. Recently, our group reported the development of the noninvasive positron emission tomography (PET) imaging agent ^68^Ga/^64^Cu-HZ20 to investigate the expression of ACE2 in the human body [10]. This PET imaging method, which allows the quantitative labelling of ACE2, is considered to be a unique tool to monitor organs infected by SARS-CoV-2.

After infection, most COVID-19 patients develop antibodies that bind to the SARS-CoV-2 RBD to neutralize the virus [11, 12]. Therefore, COVID-19 vaccines have been designed to induce the production of antibodies that bind to the spike RBD, and these vaccines play a significant role in protecting against COVID-19 development. The mechanism underlying the efficacy of these vaccines supports the effectiveness of neutralizing nanobody therapy. These vaccines are divided into 4 groups: traditionally inactivated viruses, viral vectors, nucleic acids, and viral proteins [13]. For these vaccines, some side effects are recognized as acceptable risks; however, severe adverse reactions, such as antibody-dependent enhancement and vaccine-associated enhanced respiratory disease (VAERD), must be avoided to protect patients. The safety and efficacy of vaccines show an inverse correlation. When vaccines are more effective in activating the immune system to generate the production of corresponding antibodies, viral cytopathogenicity is retained to improve the effectiveness against the spread of disease [14]. In addition, no drug has been shown to be effective effects in the medical treatment of COVID-19 patients [15]. Therefore, it is necessary to develop useful tools to study the virus.

Heavy chain-only antibodies (HcAbs) have been isolated from camels; compared to traditional monoclonal antibodies (mAb), HcAbs contain only two heavy chains and lack the CH1 chain and light chain. These heavy variable domains (VHHs) form specific interactions with antigens and produce the smallest, functional, antigen-binding fragment, termed a nanobody [16, 17]. Nanobodies have advantages such as small size, high stability, and low cost when produced via expression systems [18–20]. Moreover, nanobodies exhibit reduced aggregation and low immunogenicity in mice [21]. It has been reported that a variety of nanobodies can block the interaction of ACE2 and the SARS-CoV-2 RBD, and it has been shown that among nanobodies, Nb11-59 displays the most effective activity against SARS-CoV-2. Nb11-59 has the potential to be a preventative and therapeutic treatment against COVID-19 due to its high stability and excellent neutralization activity [22].

Serological assays, such as enzyme-linked immunosorbent assay (ELISA), are diagnostic methods based on the detection of antibodies that are produced in response to viral infection. ELISAs have the ability to provide information about active and past infections for thousands of samples. Nucleic acid amplification tests (NAATs) are sensitive methods that are used to test early viral infections, and these tests include reverse transcriptase real-time PCR (RT-qPCR) and the loop-mediated isothermal amplification-based assay (RT-LAMP). The RT-qPCR assay was considered the gold standard in the early detection of SARS-CoV-2 infection, but a point-of-care or bedside test could not be developed because of the technical complexity of RT-qPCR, which requires specialized instruments, testing reagents, and skilled operators. In addition, it is notable that the capacity to detect viral RNA by RT-qPCR almost disappeared 14 days postillness and false-negative results may also appear because of improper handling of samples. [23]. Loop-mediated isothermal amplification (LAMP) is a nucleic acid amplification-based PCR assay; however, few assays have been commercialized due to the cross reactivity and lack of sensitivity in these assays.

These difficulties highlight the need for additional detection methods. For instance, the typical features of patients with COVID-19 on initial Computed Tomography (CT) are bilateral multilobar ground-glass opacities [24]. A previous report showed that ^18^F-FDG PET could detect subclinical SARS-CoV-2 infection without obvious clinical signs in subjects, especially for patients with malignant tumors and low immunity [25]. ^18^F-FDG could reflect the pulmonary inflammatory process that occurs in the acute phase of COVID-19; however, the correlation of the pulmonary inflammatory status and the pulmonary radiological evolution or short-term clinical outcome could not be demonstrated [26]. In addition, ^18^F-FDG is not used in diagnosis of COVID-19, as the conventional agent seems to provide little benefit for chest radiography and CT [25]. PET technology is considered a progressive, noninvasive, and high-resolution/sensitive imaging method that provides real-time dynamic conditions for the whole bodies of living organisms. PET will be an excellent tool for evaluating the novel SARS-CoV-2-neutralizing nanobody and assessing its ability to target the RBD in the body. However, ^18^F-FDG is unable to detect the metabolism of the virus, and the development of a specific imaging radiotracer that binds to SARS-CoV-2 is necessary for the analysis and treatment of SARS-CoV-2.

This article provides three novel innovations: (1) development of the novel molecular probe ^68^Ga-Nb1159 based on a neutralizing nanobody and proof of its excellent ability to neutralize the RBD *in vitro* and *in vivo*, (2) demonstration of a positive correlation of ^68^Ga-Nb1159 and RBD residue levels in the body with PET technology, and (3) development of a novel method to evaluate the metabolism of the neutralizing nanobody Nb11-59. Based on this study, this method is expected to be useful for the evaluation of other neutralizing nanobodies that target the SARS-CoV-2 RBD and the determination of the location of the RBD in the body to guide precision therapy during SARS-CoV-2 infection.

**Scheme 1:**
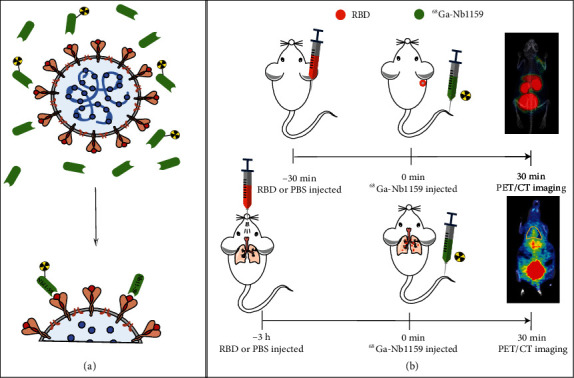
Schematic illustration of ^68^Ga-Nb1159 binding to the RBD and PET imaging. (a) ^68^Ga-Nb1159 and the neutralizing nanobody Nb11-59 may bind to the SARS-CoV-2 RBD to block the interaction of the SARS-CoV-2 RBD and ACE2 receptor. (b) Schematic illustration of the mice model of treatment with the SARS-CoV-2 RBD and timeline for PET/CT imaging after ^68^Ga-Nb1159 i.v. injection.

The concept graph is shown in Scheme 1(a).

## 2. Results

### 2.1. Biopanning and Identification of SARS-CoV-2 Spike RBD-Specific Nanobodies

**Figure 1:**
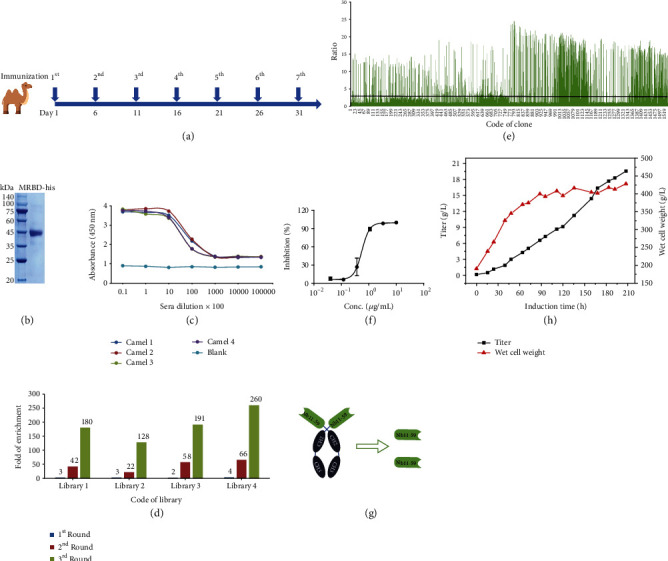
Immunization, biopanning, and identification of SARS-CoV-2 spike RBD-specific nanobodies. (a) The schedule of camel immunization. (b) SDS-PAGE analysis of SARS-CoV-2 spike RBD-His. (c) The titer of antisera was evaluated after immunization. (d) The details of enrichment in each round of biopanning. (e) All the ratio values of the colonies identified by PE-ELISA. Evaluation of neutralizing and the process development of neutralizing antibody (f–h). (f) 50% ND_50_ of Nb11-59 was calculated by mean value ± SD. (g) Schematic diagram shows the structures of Nb11-59. (h) The yield and wet cell weight of Nb11-59 in fermentation tank with continuous induction times.

Four camels were immunized with the SARS-CoV-2 spike RBD seven times according to the schedule shown in Figure 1(a). After purification, the SARS-CoV-2 spike RBD fused to a His tag was characterized by SDS-PAGE, as shown in Figure 1(b); the results show that the highly pure antigen had a molecular size that was consistent with the theoretical values.

After the seventh immunization, the titers in the antisera of the four camels were evaluated by ELISA, and the results are shown in Figure 1(c). All the titers reached 1.0 × 10^5^, which indicated strong immune stimulation.

After successful immunization, phage display libraries were constructed by PBL isolation, RNA extraction, cDNA preparation, VHH gene amplification, ligation, and transfection. The sizes of the four libraries were all calculated to be approximately 1.0 × 10^9^ colony-forming units (CFU), and the correction insertion rates were higher than 90%, suggesting high quality and good diversity of the libraries.

Phage display technology was used to screen SARS-CoV-2 spike RBD-specific VHHs. As shown in Figure 1(d), after three rounds of biopanning, the fold enrichment values all reached more than 100, suggesting effective enrichment. Then, a total of 1600 colonies were randomly selected from the second and third rounds to evaluate the ability to bind to the SARS-CoV-2 spike RBD via a PE-ELISA, and 690 colonies were defined as positive colonies with a binding ratio greater than 3 (Figure 1(e)). Based on the alignment of amino acid sequences, 381 distinct positive molecules were retained after the removal of repeat sequences.

### 2.2. The Identification and Development of Neutralizing Nanobodies

Functional candidates were identified and characterized using the following procedures. First, 229 of 381 molecules were selected based on their cross reactivity to 8 different SARS-CoV-2 RBD mutants. Second, 32 candidates with the ability to block the spike RBD and bind to ACE2 were verified based on a blocking assay. Third, 7 candidates were identified based on the consideration of molecular properties, blocking activity, and other characteristics. Importantly, the ability of the 7 candidates to neutralize authentic SARS-CoV-2 was determined *in vitro*. Nb11-59 exhibited the best neutralizing ability in a plaque reduction neutralization test (PRNT), with an inhibitory rate higher than 60%; additionally, the ND_50_ was 0.55 *μ*g/mL, as determined by a decrease in phage count (Figure 1(f)).

The monovalent VHH structure was selected as the final molecular format of Nb11-59 (Figure 1(g)). Considering future application, low cost, and large-scale production, the *Pichia pastoris* system was chosen for expression and fermentation. As shown in Figure 1(h), the titers in the fermentation supernatants reached approximately 20 g/L, and the purity was 99.36% according to SEC-HPLC analysis. Fortunately, we almost completely accomplished the preliminary drug-ability analysis. Nb11-59 was successfully delivered by nebulization. Generally, Nb11-59 is a good candidate therapeutic antibody against COVID-19, and it is worthy of further research in the medical, imaging, and diagnostic fields.

### 2.3. *In Vitro* Characterization of ^68^Ga-Nb1159

**Figure 2:**
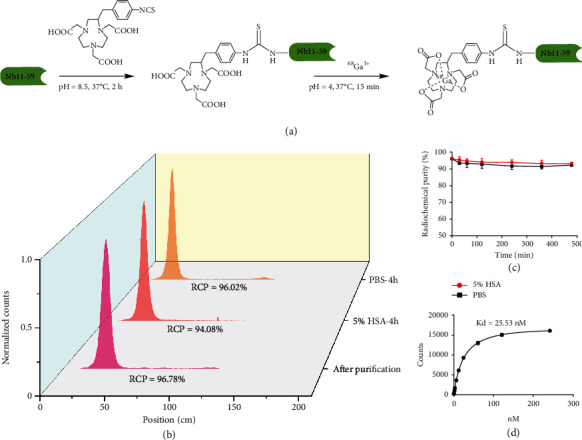
*In vitro* characterization of ^68^Ga-Nb1159. (a) Schematic illustration of the synthesis of the ^68^Ga-Nb1159 probe. (b) The normalized radiochemical purity of unpurified and purified ^68^Ga-Nb1159, and the stability of ^68^Ga-Nb1159 at 4 h. (c) Stability analysis of ^68^Ga-Nb1159 over time in 0.01 M PBS and 5% HSA at 37°C. (d) Binding affinity assay of ^68^Ga-Nb1159 to RBD of SARS-CoV-2.

The procedure of ^68^Ga-Nb1159 radiosynthesis is shown in Figure 2(a); the radio-TLC showed that the nondecayed radiochemical yield of ^68^Ga-Nb1159 was 49.48 ± 3.12%, and it had a radiochemical purity of >95% (Figure 2(b)) and a specific activity of 2.74-10.99 MBq/nmol. Both the *in vitro* and *in vivo* stabilities of ^68^Ga-Nb1159 were determined with radio-TLC.

In terms of *in vitro* stability, the radiopharmaceutical assessment revealed excellent tracer stability after incubation in 5% HSA and 0.01 M PBS; the tracer remained stable for 8 h, and its radiochemical purity was over 90% (Figure 2(c)). In terms of *in vivo* stability, the radiochemical purity of ^68^Ga-Nb1159 in urine basically did not vary, but the RCP in blood slightly decreased (Fig. S1a).

A binding study was performed to determine the binding affinity of ^68^Ga-Nb1159 to RBD. Data were analyzed using Graph Pad Prism Software to determine the *K*_*d*_. The *K*_*d*_ value was 25.53 nM according to a one-site binding model (Figure 2(d)). The results showed that ^68^Ga-Nb1159 specifically binds to RBD *in vitro*.

### 2.4. Biodistribution Study and Pharmacokinetics Study

**Figure 3:**
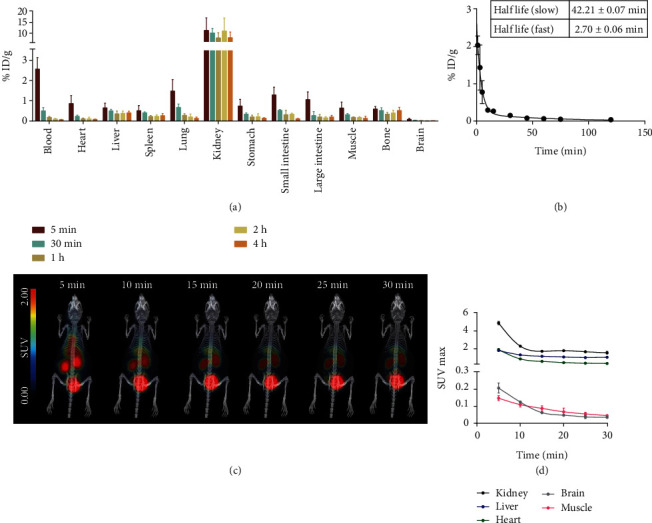
*In vivo* characterization of ^68^Ga-Nb1159. (a) Biodistribution of ^68^Ga-Nb1159 in KM mice at different time points postinjection (*n* = 3, 20 *μ*Ci per mouse i.v.). (b) Pharmacokinetics of ^68^Ga-Nb1159 in KM mouse. (c) Dynamic preclinical PET imaging of ^68^Ga-Nb1159 in KM mouse within 30 min. (d) SUV max values in naive organs of dynamic Preclinical PET imaging in Figure 3(c).

In naive Kunming (KM) mice, ^68^Ga-Nb1159 showed rapid clearance in the kidneys, and low uptake in other organs (Figure 3(a)). ^68^Ga-Nb1159 accumulated at high levels in the kidneys with uptake values of 11.25 ± 5.89 ID%/g at 5 min, and the values remained consistent from 5 min to 4 h. Subsequently, uptake was observed in the other organs, including the heart, liver, spleen, lung, and brain, and it decreased gradually. At 30 min, the uptake value of the kidney (10.14 ± 2.15 ID%/g) was higher than that of other organs or tissues, including the lung (0.67 ± 0.16 ID%/g), small intestine (0.53 ± 0.04 ID%/g), bone (0.52 ± 0.14 ID%/g), liver (0.50 ± 0.089 ID%/g), and blood (0.50 ± 0.16 ID%/g).

A pharmacokinetic study of ^68^Ga-Nb1159 proved that the tracer was rapidly cleared from the mouse body. As concluded from pharmacokinetics analysis (Figure 3(b)), the fast half-life of ^68^Ga-Nb1159 was 2.70 ± 0.06 min and the slow half-life was 42.21 ± 0.07 min, respectively.

### 2.5. Preclinical PET Imaging Study

Dynamic PET imaging of naive KM mice showed that ^68^Ga-Nb1159 was rapidly cleared from the mouse body and mainly accumulated in the kidneys and bladder, with low uptake in the liver (Figure 3(c)). Although the liver uptake was similar to that of the spleen and lung in Figure 3(a), the density of liver was heavier compared to the spleen and lung. So, spleen and lung uptakes were much lower than that of the liver in overall PET imaging (Figure 3(c)). The dynamic SUVmax curve showed that the kidneys exhibited high uptake of the tracer, followed by the liver (Figure 3(d)). The maximum single-voxel standardized uptake value (SUVmax), the standard nuclear medicine metric, was chosen to show the probe accumulation in tissues or organs, through measuring the maximum voxel value in a volume of interest (organs) standardized to patient mass and administered activity. The SUVmax definition was in Supplementary materials. From PET imaging, the accumulation of ^68^Ga-Nb1159 in the organs mentioned above was highest at 5 min p.i. and decreased with time. Furthermore, the SUVmax remained consistent after 20 min p.i. Subsequently, static PET imaging of KM mice was performed at 1 h and 2 h p.i. (Fig. S2a). The scan results were similar to those of dynamic imaging at 30 min. The results showed that high levels of tracer accumulated in the kidneys and bladder.

**Figure 4:**
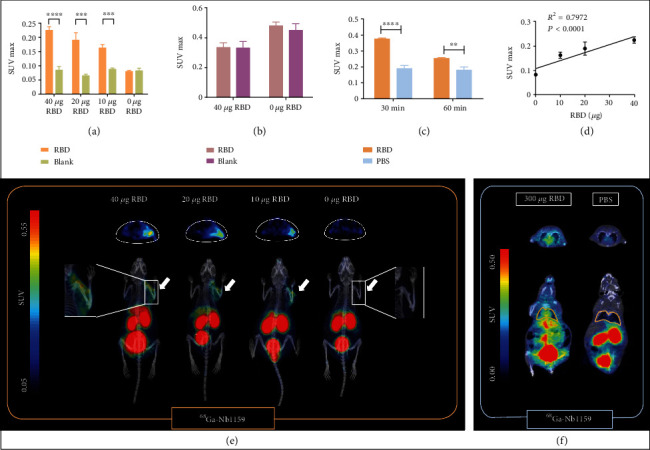
Preclinical PET imaging and analysis of mice model treated with the RBD. (a) Comparison of the SUVmax in Figure 4(e) between RBD-injected muscle and contralateral muscle (control group). (b) Comparison of SUVmax in Fig. S1c between RBD-injected muscle and contralateral muscle (control group) after ^18^F-FDG injection i.v. (c) Comparison of SUVmax in lungs from Figure 4(f) at 30 min and 60 min after ^68^Ga-Nb1159 injection i.v. ^∗∗^*p* < 0.01,  ^∗∗∗^*p* < 0.001, and^∗∗∗∗^*p* < 0.0001. (d) The correlation of SUVmax and RBD amount. (e) Preclinical PET imaging of ^68^Ga-Nb1159 i.v. injected into KM mice after subcutaneous injection of the RBD. The white arrow indicates the subcutaneous injection of the RBD. (f) Preclinical PET imaging of ^68^Ga-Nb1159 i.v. injected into KM mice at 60 min after intrapulmonary injection of the RBD or 0.01 M PBS.

Preclinical PET imaging of KM model mice that were injected with the RBD in the right shoulder region indicated that ^68^Ga-Nb1159 exhibited specificity for the RBD *in vivo* (Figure 4(e)). This probe could locate the region and reveal the distribution of the RBD. Compared with that in the contralateral region, the accumulation of ^68^Ga-Nb1159 in the RBD-injected regions increased significantly (40 *μ*g, 20 *μ*g, and 10 *μ*g) in terms of both imaging (Figure 4(e)) and SUVmax (Figure 4(a)). The SUVmax continually increased as the amount of RBD increased (Figure 4(a)). Furthermore, the SUVmax showed a positive correlation with the RBD amount (Figure 4(d)). This correlation indicated that some evaluation of the effect of therapeutic antibody treatment should be conducted. In addition, the imaging results were similar to naive KM mouse imaging results, including high tracer uptake in the kidney, bladder, and liver; and the SUVmax of other organs as shown in Figure S1b.

When ^18^F-FDG was injected to detect the RBD *in vivo*, the uptake in the RBD-injected region was not different from that in the contralateral region (Fig. S1c), and the SUVmax in the RBD-injected region was not significantly different from that in the control region (Figure 4(b)). The SUVmax of other organs is shown in Fig. S1d.

To further study the specificity of ^68^Ga-Nb1159 for RBD, other mouse models in which the RBD and PBS were separately inoculated into the lungs were utilized for PET scanning. The imaging results indicated that two mice had obvious differences after the injection of ^68^Ga-Nb1159. The outline of the lungs was drawn at 60 min (Figure 4(f)) and 30 min p.i. (Figure S2b). The uptake into the lungs was more obvious in the RBD model. The SUVmax was significantly elevated in the lungs of mice injected with the RBD at 30 min (0.38 ± 0.01) and 60 min p.i. (0.25 ± 0.01, Figure 4(c)). In addition, the whole body and vitro PET imaging of mice model with RBD revealed obvious uptake of ^68^Ga-Nb1159 (Fig. S3), The biodistribution in the left and right lungs is shown in Fig. S3d. These results suggested that ^68^Ga-Nb1159 exerted a better diagnostic effect for the RBD and confirmed that the neutralizing nanobody Nb11-59 possesses the ability to neutralize against SARS-CoV-2 *in vivo*.

### 2.6. Initial Evaluation of the Pharmacodynamic Effects of the Agent

**Figure 5:**
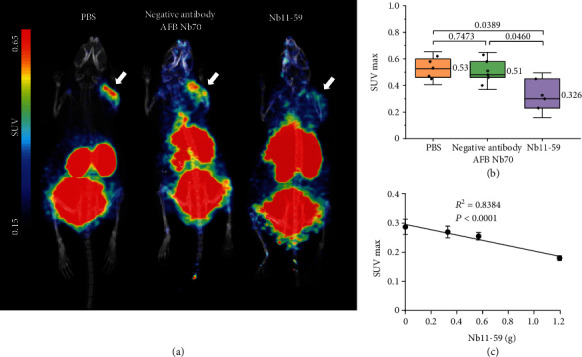
Preclinical PET imaging and analysis of mice coinjected with PBS, AFB Nb70 and Nb11-59. (a) Preclinical PET imaging of ^68^Ga-Nb1159 i.v. coinjected with PBS, a negative control nanobody and Nb11-59 in KM mice after subcutaneous injection of the RBD. The white arrow indicates the subcutaneous injection of the RBD. (b) Comparison of SUVmax in three groups of mice coinjected with PBS (*n* = 4), a negative control nanobody (*n* = 5, 1 mg per mouse i.v.), and Nb11-59 (*n* = 3, 1 mg per mouse i.v.). (c) The correlation of SUVmax and Nb11-59 amount coinjected with the agent.

Nb16-68 displayed excellent activity against SARS-CoV-2 [22]. The nanobody Nb70, which was detected with aflatoxin B1, was used as a negative control nanobody [27]. The neutralizing nanobodies Nb11-59 and Nb16-68 were considered positive control therapeutic nanobodies in this experiment because of their ability to bind to the SARS-CoV-2 RBD. To determine the pharmacodynamic effects of other antibodies that bind to the SARS-CoV-2 RBD and are used for the treatment of virus infection, ^68^Ga-Nb1159 was coinjected with Nb11-59, the negative control nanobody, or normal PBS. Then, the SUVmax values calculated from the PET images revealed the pharmacodynamic effects of the antibodies, which could be used to prevent further spread of the pandemic. The PET images are shown in Figure 5(a). Mice injected with Nb11-59 showed reduced uptake in the right shoulder (SARS-CoV-2 RBD-injected region). A better treatment effect means a lower PET imaging-based SUVmax in this analysis. The mean SUVmax was 0.32 in the group that was coinjected with Nb11-59. In comparison, the SUVmax was 0.53 and 0.51 in the groups that were coinjected with the negative nanobody and PBS, respectively (Figure 5(b)). According to PET imaging, the peak SUVmax was 0.62 in the PBS group, which was similar to that in the negative nanobody group (0.63) and exceeded that in the Nb11-59 group (0.45). In addition, when we injected different amounts of Nb11-59 with this agent, the SUVmax showed a dose-dependent decrease (SUVmax of 0.29, 0.27, 0.26, and 0.18 when Nb11-59 was injected at 0 mg, 0.33 mg, 0.57 mg, and 1.20 mg, respectively), as shown in Figure 5(c). PET imagings are shown in Fig. S3a. Similarly, PET imaging and SUVmax analysis (Fig. S3b) of the mice coinjected with the neutralizing nanobody Nb16-68 agreed with the observed beneficial effects. The results demonstrated that the pharmacodynamic effects of the antibody targeting the SARS-CoV-2 RBD could be detected with the agent ^68^Ga-Nb1159. Antibodies with excellent therapeutic effects should exhibit reduced uptake and SUVmax values.

## 3. Discussion

The rapid spread of COVID-19, which is caused by SARS-CoV-2 continues worldwide. As of May 2022, over 520 million cases and just under 6.27 million related deaths have been reported worldwide according to the WHO. The development of additional diagnostic tools and further analysis of the disease are necessary. SARS-CoV-2 infection in humans relies on the RBD of SARS-CoV-2 binding to human ACE2.

To date, vaccines are considered the primary method of prevention, but the development of effective vaccines faces several challenges, such as the need to identify specific features and the virulence abilities of newly mutated viruses. In addition, there is public concern that live attenuated and inactivated vaccines are likely to revert to virulence. Other challenges exist in the large-scale production of pure and stable vaccines [28]. The treatment of COVID-19 focuses on pharmacological therapies (such as remdesivir, fostamatinib, and chloroquine), anti-SARS-CoV-2 antibody cocktails, and other treatments, such as convalescent plasma (CP) [29]. There are some difficulties associated with these treatments. The concomitant use of remdesivir and chloroquine may reduce the effect of remdesivir. The rate and likelihood of recovery may decrease in patients who use both drugs [29]. Early CP treatment is able to prevent clinical deterioration, but this treatment has the disadvantage of limited supply of plasma from survivors. Monoclonal antibody therapy helps patients relieve the symptoms that are caused by the virus; however, this approach requires extremely high doses of monoclonal antibodies. In addition, it is difficult to quickly produce traditional monoclonal antibodies at low cost [30].

The RBD of SARS-CoV-2 has two main conformations, the “up” and “down” conformations. The “up” conformation exhibits feature that are more easily bound by ACE2 and most nanobodies than the “down” confirmation [31]. Therefore, neutralizing nanobody treatment shows great potential for curing COVID-19. The characteristics of nanobodies, such as high stability, small structure, and their high specificity, make them ideal for purification. In addition, the stability of nanobodies enables them to be nebulized and directly delivered to the lungs. Furthermore, the activity of nanobodies remains stable during long-term storage [30]. It has been reported that nanobodies can neutralize SARS-CoV-2, are effective against emerging variants, and are resistant to mutational escape [32].

Previous research has identified an excellent neutralizing nanobody, Nb11-59. It not only exhibits the advantages mentioned above but also effectively recognizes the wild-type RBD and eight kinds of RBD mutants from mutated viruses. The ND_50_ of Nb11-59 was 0.55 *μ*g/mL, which demonstrated that the nanobody could inhibit the replication of SARS-CoV-2 *in vitro* [22]. The amino acid sequence, SDS-PAGE data, and Matrix-Assisted Laser Desorption Ionization-Time of Flight Mass Spectrometry (MALDI-TOF-MS) Charts of Nb11-59 and NOTA-Nb1159 are listed in Fig. S7 of Supplementary Materials. We confirmed the conjugation of NOTA on the nanobody through MALDI-TOF-MS. The different molecular weight between the NOTA-Nb1159 (13839.099) and Nb11-59 (13406.962) was close to the molecular weight of NCS-Bz-NOTA (450.51). The result demonstrated that one NOTA was conjugated on the Nb11-59.

The evaluation of nanobodies against SARS-CoV-2 may reveal the mechanisms underlying the processing and alteration of the virus, thereby facilitating observation of treatment effects, evaluation of responses to prevention and treatment strategies, and development of more reliable vaccines and drugs. However, the traditional evaluations of neutralizing nanobody metabolism and the RBD primarily include biolayer interferometry (BLI) and plaque reduction neutralization test (PRNT) technology, based on previous studies. These methods are expensive, laborious, and time-consuming, and it is difficult to evaluate neutralizing nanobodies *in vivo*. Evaluation of Nb11-59 by PET technology had the following advantages: (1) it is considered a progressive, noninvasive, and high-resolution/sensitivity visual imaging method, (2) it provides real-time information about the dynamic conditions of the whole bodies of living organisms, and (3) it predicts responders or can be used to monitor responses to therapies or vaccines. Therefore, PET will be an excellent tool to evaluate the novel SARS-CoV-2-neutralizing nanobody and detect its ability to target the RBD in the body.

This study was designed to evaluate ^68^Ga-Nb1159 *in vitro* and *in vivo* and confirmed the conceptual assumption that ^68^Ga-Nb1159 can distinguish the localization and distribution of the SARS-CoV-2 RBD *in vivo*. The radiochemical yield and specific activity of ^68^Ga-Nb1159 are relatively low compared with those of other nanobodies radiolabeled with ^68^Ga^3+^ [33–35]. The possible reason may be related to the amount of product retained in the PD-10 column, but other reasons must be considered and verified. The method of radiosynthesis will be optimized in the future.

The *in vitro* experiments revealed the excellent stability (RCP > 94%) and significant binding ability of ^68^Ga-Nb1159 to the SARS-CoV-2 RBD (*K*_*d*_ = 25.53 nM), and the *K*_*d*_ value is consistent with a previous study [22]. The *in vitro* findings indicate that the radiotracer deserves further investigation. The stability data from blood is truly indicated the in vivo decomposition of ^68^Ga-Nb1159, and the result from urine samples further confirmed the similar metabolite was observed in both samples. Our previously research also indicated that ^68^Ga-NOTA-peptides might be unstable. However, the free ^68^Ga, ^68^Ga-NOTA, and ^68^Ga-NOTA-conjugated decomposed proteins of ^68^Ga-NOTA-Nb1159 have no or low affinity to the targets, and they are rapidly excreted through urine, which might slightly influence the contrast ratio of ^68^Ga-Nb1159 over lesions (Figure 5(a)). It is worthy to characterize these metabolites so that we can further develop more stable version for clinical applications.

In biodistribution and pharmacokinetics studies in KM mice, ^68^Ga-Nb1159 showed rapid clearance *in vivo* and accumulation in the kidney, with a low background signal. These results coincided with the inherent features of nanobodies, such as their small size and rapid metabolism in the kidney. On the other hand, this conclusion was supported by the results of dynamic PET imaging of naive KM mice. Obvious high uptake in the kidney and bladder was observed, and the liver also exhibited tracer accumulation although to lower extents than the kidney. The radionuclide Ga^3+^ might separate from the radiotracer and lead to more radioactivity accumulation.

A previous study revealed that COVID-19 patients exhibited clearly elevated expression levels of cytokine and inflammation-related genes and indicated that monocytes, T cells, and megakaryocytes might be major causes of inflammation [36]. Other inflammation-associated PET imaging probes, such as ^18^F-FDG or [^18^F]DPA-714, might be used as control probes since inflammation is significant in COVID-19 infection. Preliminary studies have reported PET imaging of COVID-19 patients with ^18^F-FDG [37–39], and ^18^F-FDG PET could detect subclinical COVID-19 without obvious clinical signs in subjects [25]. Therefore, ^18^F-FDG was chosen as a control tool to evaluate the distribution of the SARS-CoV-2 RBD with ^68^Ga-Nb1159. PET imaging showed that ^68^Ga-Nb1159 was more specific than ^18^F-FDG in mice injected with the SARS-CoV-2 RBD. Compared with the nonspecific imaging ability of ^18^F-FDG, ^68^Ga-Nb1159 exhibited potential not only for monitoring the distribution of SARS-CoV-2 in real time but also for evaluating the infection status. This dynamic monitoring method was used to assess individual patients' conditions.

The low uptake of the tracer in other organs and muscle made the distribution of the radiotracer clear, which enabled PET imaging of mice injected with theSARS-CoV-2 RBD to confirm the binding ability of ^68^Ga-Nb1159. The SUVmax in the RBD-injected regions and the amount of RBD exhibited an obvious positive correlation (*R*^2^ = 0.7972, *P* < 0.001). This finding means that the radiotracer may be a quantitative analytic tool. Two other groups in Fig. S5 in Supplementary Materials. They showed the similar tendency.

On the other hand, the SUVmax (RBD = 40 *μ*g) of the injected region was approximately triple that of the contralateral muscle and then decreased to 1.85-fold and 0.97-fold as the amount of RBD decreased to 10 *μ*g and 0 *μ*g, respectively. The mice injected with the RBD in the lung exhibited higher uptake (approximately twofold) than the mice administered PBS in the lung. For analysis *in vitro*, pulmonary uptake with RBD was approximately double that with PBS. The results in models with different RBD localization demonstrated that the probe could convincingly detect the SARS-CoV-2 RBD. Other agents, such as [^18^F]DPA-714, which was developed to monitor neuroinflammation, also showed approximately triple uptake in the inflammatory region compared with that in the naive region [40]. The bacterial PET agent D-[^11^C]ala accumulated in the tail where *Staphylococcus aureus* was injected. ROI analysis of PET imaging showed a 3.3-fold higher signal at the infected site than in background tissue [41]. The ratio of ^68^Ga-Nb1159 in RBD-injected muscle and naive muscle is powerful proof that it can be used to visualize the distribution of the SARS-CoV-2 RBD *in vivo*.

The dose-dependent change in the SUVmax value after the coinjection of the nanobody Nb11-59 and the obvious contrast in PET imaging of different mice (coinjected with Nb11-59, negative control antibody, and PBS) showed that ^68^Ga-Nb1159 was able to detect the pharmacodynamic effects and therapeutic effect exerted by other antibodies to some extent.

The SUVmax of mice (40 *μ*g RBD) injected with PBS and ^68^Ga-Nb1159 in Figure 5(b) were higher than those in Figure 4(a). The result was attributed to the different collected times. In Figure 5, they were collected at 20 min after ^68^Ga-Nb1159 administrations and a′ (the decay-corrected amount of injected radiolabeled tracer) is different indicating various intervals due to the compact scan arrangement. The details are explained in Supplementary Materials. In order to amend this time-dependence flaw, we compared the Target-to-Nontarget activity ratio (T/NT), which is a ratio between target and nontarget organs or tissues (Fig. S6, in Supplementary Materials). In the group (SUVmax was 0.25, Figure 4(a)), the T/NT values were 2.75 ± 0.43. In the other group (SUVmax was 0.53, Figure 5), the T/NT values was 3.21 ± 0.46. The two group values did not have significantly different. The experimental results suggested that we developed a novel method to evaluate nanobodies with PET technology. This method might visually reveal the metabolic pathways of SARS-CoV-2 in the body and can be used to detect the treatment effects or monitor the response to therapies in real time. The result was promising, so further studies are needed in the future.

This study was based on the use of a radionuclide to label nanobodies that can neutralize SARS-CoV-2 in order to evaluate the features of the nanobody *in vivo* and *in vitro* for the first time. It is worth mentioning that we accomplished two goals, including the evaluation of metabolism and the verification of the ability of Nb11-59 to target the SARS-CoV-2 RBD in the body through radiolabeling and PET methods; these approaches were previously limited due to the lack of research and development of radio-nanobody agents.

There is still a limitation of this research. We used the RBD of SARS-CoV-2 to replace the real virus because of the limitations associated with purchasing the virus. Although previous research has concluded that an infected person carries approximately 1 billion to 100 billion virions and that the total viral mass is at most 0.1 mg [42], this study was unable to estimate the mass of the virus based on the RBD. We therefore could not estimate the total number of viruses in infected humans based on the SUVmax value obtained by PET imaging.

On the other hand, clinical research should be conducted to evaluate the metabolic situation of the virus and the effects of treatment in SARS-CoV-2-infected and recovered persons.

^68^Ga-Nb1159 relies on remarkable specific binding on the SARS-CoV-2 RBD and has promising research prospects in the diagnosis and analysis of COVID-19. In addition, further studies of the probe are also necessary, including preclinical evaluation with SARS-CoV-2, evaluation of the pharmacodynamic effects of therapeutic antibodies, and clinical research on asymptomatic SARS-CoV-2 infection and patients who have recovered from COVID-19.

In general, these experiments were conducted to evaluate the characteristics of ^68^Ga-Nb1159 from many perspectives. In this study, a NOTA-NCS conjugated to a neutralizing nanobody, Nb11-59, was prepared and radiolabeled with ^68^Ga^3+^ to track the SARS-CoV-2 spike RBD. ^68^Ga-Nb1159 production can be achieved with high radiolabeling yield and exhibits excellent stability. In addition, this novel PET targeting probe exhibited a fast metabolic rate and specifically recognized the RBD *in vivo*. Furthermore, this promising approach for visually monitoring the distribution of SARS-CoV-2 and assessing the treatment effects of therapeutic antibodies in humans in real time.

## 4. Materials and Methods

### 4.1. General

All reagents were obtained from commercial vendors and used without and further purification. Phosphate-buffered saline (PBS, 0.01 M. pH 7.4) and 2-propanamine, N-ethyl-N-(1-methylethyl) (DIEA) were purchase from Aladdin, Shanghai, China. Nanobodies Nb11-59, Nb16-68, and Nb70 were provided by Shanghai Novamab Biopharmaceuticals Co., Ltd., Shanghai, China. Sodium acetate was from Alfa Aesar, Thermo Fisher Scientific, United Kingdom. The NCS-Bz-NOTA was purchased from Beijing Innochem Science & Technology Co., Ltd., Beijing. PD-10 columns were from Cytiva, China (catalog # 17371260). The product purity was determined using Radio-TLC (AR 2000, Bioscan, USA). The PET/CT imaging studies of small animals were performed on the Mira PET/CT of PINGSENG Healthcare Inc. (Shanghai, China). The ^68^Ge-^68^Ga generator was purchased from ITM Co., Ltd., Germany.

### 4.2. Camel Immunization

SARS-CoV-2 spike RBD (Arg319-Asn532) was expressed in HEK293F mammalian cells with a His tag at the N-terminus and purified by Ni-NTA affinity chromatography. The purified SARS-CoV-2 spike RBD was mixed with Freund's adjuvant for camel immunization. Four healthy Bactrian camels were immunized for seven times; then enzyme-linked immunosorbent assay (ELISA) was employed to test the titer of antiserum. Afterwards, 100 mL peripheral blood from the camels was collected for phage display library construction. All camel experiments were performed in compliance with Ethics Guidelines approved by Shanghai Science and Technology Committee (STCSM).

### 4.3. Phage Display Library Construction

The peripheral blood lymphocytes (PBLs) were isolated, and total RNAs were amplified by RNA extraction kit. The cDNAs were reverse transcripted, and VHHs were prepared by two-step nested PCR. The purified VHH fragments were subcloned into phage-display phagemid pMECS after digestion by restriction enzymes. The quality of libraries was evaluated by library size and VHH fragment insertion rate.

### 4.4. Specific Nanobody Nb11-59 Isolation

The SARS-CoV-2 spike RBD-specific nanobodies were screened by phage display technology. After three consecutive rounds of biopanning, around 1600 individual colonies from the four libraries were randomly picked for positive candidate identification by performing periplasmic extract ELISA (PE-ELISA).

### 4.5. Synthesis, Radiolabeling, and Quality Control of ^68^Ga-Nb1159

The nanobody Nb11-59 was conjugated to NCS-Bz-NOTA at a molar ratio of 1 : 5 at pH 8.5 and temperature of 37°C for 2 h. The product, NOTA-Nb1159, was purified through PD-10 size-exclusion columns. The concentrations of nanobody conjugated NOTA were measured by a NanoDrop 2000 UV-visible Spectrophotometer.

For ^68^Ga radiolabeling, ^68^GaCl_3_ solution (1.5 mL, 267 MBq) obtained from ^68^Ge-^68^Ga generator, sodium acetate (97 *μ*L, 1 M), and Nb11-59 (250 *μ*L, 1.2 mg/mL) were added and reacted in a single kit vial at 37°C for 15 min. After reaction, the mixture was purified through PD-10 columns (pretreated with 25 mL 0.01 M PBS), and the final product ^68^Ga-Nb1159 was monitored by radio-TLC with saline solution containing 4 mM of ethylenediaminetetraacetic acid (EDTA).

### 4.6. *In Vitro* and *In Vivo* Stability Study

*In vitro* stability of ^68^Ga-Nb1159 was determined in 0.01 M PBS and 5% human serum albumin (HSA) at 37°C. At six different time points (30 min, 60 min, 120 min, 240 min, 360 min, and 480 min), 4 *μ*L (0.3 MBq) mixture was taken out and tested for the radiochemical purity by radio-TLC. For *in vivo* stability assessment, Kunming mice (female, 18-20 g, *n* = 3) were intravenously administered approximately 37 MBq of ^68^Ga-Nb1159. The urine and blood were collected and analyzed at 5 min, 30 min postinjection, and the radiochemical purity of the tracer in these above samples with radio-TLC.

### 4.7. Enzyme-Linked Immunosorbent Assay (ELISA)

To determine the binding potency between the nanobody Nb11-59 and the SARS-CoV-2-RBD-His, it was dissolved in 0.01 M PBS to 1 *μ*g/mL, and 100 *μ*L RBD was coated on the 96-microtiter plate per well at 4°C, overnight. The antigen solution was removed and washed 5 times with PBST (containing 0.05% T-20). To block other nonspecific sites, 100 *μ*L 5% powdered milk was added at 37°C for 2 h, then discarded, and washed with PBST. The antigen was exposed to increasing concentrations of ^68^Ga-Nb1159 (0.37 KBq/mL to 7400 KBq/mL) with 100 *μ*L/well for 1 h at 37°C and then still washed five times with PBST. Finally, each incubation well was cut and analyzed by a fully automatic gamma counter.

### 4.8. Small Animal Biodistribution and Pharmacokinetics Study

For the animal biodistribution study, KM mice (female, 18-20 g) were divided into 5 groups (*n* = 3) randomly and injected with ^68^Ga-Nb1159 (1.11 MBq per mouse i.v.). The mice were sacrificed after anaesthetization at 5 min, 30 min, 1 h, 2 h, and 4 h postinjection. The heart, liver, spleen, lung, kidney, stomach, bone, muscle, blood, and other gastrointestinal organs were removed, weighted, and counted with a gamma counter; meanwhile, 10 samples of 1% injected dose of ^68^Ga-Nb1159 were counted as a standard control. The results were expressed as the percent uptake of injected dose per gram of tissue (% ID/g) and presented as the mean ± SD.

The pharmacokinetics study of ^68^Ga-Nb1159 was accomplished using KM mice (female, *n* = 4), which was injected with 3.7 MBq of the tracer intravenously. At various predetermined intervals (1 min, 3 min, 5 min, 10 min, 15 min, 30 min, 45 min, 60 min, 75 min, and 120 min), a small portion of blood was acquired through the orbital vein, and each blood sample was weighted before counted using a gamma counter. The results were calculated as the percentage injected dose per gram (% ID/g, mean ± SD).

### 4.9. Preclinical PET/CT Imaging in Naive KM Mouse

The naive KM mouse was sedated with isoflurane anesthesia (2-3%, 1 L/min oxygen) and placed on a heating bed to perform preclinical PET/CT imaging scanner (SuperNova, PINGSENG Healthcare, China), then injected with 200 *μ*L of ^68^Ga-Nb1159. The dynamic PET images were acquired over a period of 30 min (5 min/frame). Regions of interest (ROIs) were drawn on the CT images and further mapped on PET. The SUVmax of organs were collected and calculated to a dynamic curve. The static PET scans of ^68^Ga-Nb1159 were obtained at 60 min and 120 min postinjection. In addition, the ratio of ^68^Ga to nanobody Nb11-59 was 0.02% if we assumed that one Nb11-59 chelated one radionuclide ^68^Ga. The computational process was showed in supplement.

### 4.10. Mouse Models with the RBD and Preclinical PET Imaging

As Scheme 1(b) shows, to test the specificity of the nanobody Nb11-59 for SARS-CoV-2, Kunming (KM) mice (female, 18-20 g) were injected with different amounts of the SARS-CoV-2 spike RBD in PBS (40 *μ*g, 20 *μ*g, or 10 *μ*g) in the right shoulder region. As a comparison, 0.01 M PBS was injected into the same region of other mice. The RBD and PBS were allowed to disseminate for 30 min, and then, the mice were intravenously (i.v.) injected with ^68^Ga-Nb1159 and imaged according to the above protocol using preclinical PET after 30 min. As a comparison, the above mice injected with the RBD in the shoulder region at 40 *μ*g and 0 *μ*g were evaluated by the most commonly applied PET agent, ^18^F-FDG. Then, the maximum standardized uptake value (SUVmax) of the RBD-injected region and contralateral region was compared.

A critical experiment was performed to determine whether Nb11-59 could provide specificity to SARS-CoV-2 at other organs *in vivo*. KM mice (female, 18-20 g) were inoculated intratracheally with 300 *μ*g of RBD (in 100 *μ*L of PBS) or 0.01 M PBS (100 *μ*L) and then, after anesthetization, intraperitoneally injected with 0.2 mL of 4% chloral hydrate. This method has been described previously [43]. Then, ^68^Ga-Nb1159 was injected i.v. after 3 h; preclinical PET imaging scans were obtained under the same protocol guidance at 30 min and 60 min postinoculation (p.i.). The different SUVmax of the lungs was compared between RBD-injected and 0.01 M PBS-injected mice. In addition, the lungs with RBD (75 *μ*g) and PBS were removed at 60 min p.i. Next, the left lung and right lung were conducted the preclinical PET imaging *in vitro* and weighted and counted with a gamma counter.

### 4.11. Initial Evaluation of the Pharmacodynamic Effects of the Agent

This protocol was similar to that used in the previous steps. KM mice (female, 25-30 g) were injected with the SARS-CoV-2 spike RBD in PBS (40 *μ*g) in the right shoulder region. After 30 min, the mice were coinjected with PBS (*n* = 4), the negative control nanobody AFP Nb70 (*n* = 5, 1 mg per mouse i.v.), or Nb11-59 (*n* = 3, 1 mg per mouse i.v.). Then, PET images of the mice were obtained according to the above protocol using preclinical PET. In addition, the SUVmax of the RBD in the three groups was determined and compared. In addition, the SARS-CoV-2 RBD-injected mice was coinjected with different amounts of Nb11-59 and other neutralizing nanobody (Nb16-68), and PET imaging and similar analysis were performed.

## Data Availability

All data in this study are available from the corresponding authors upon reasonable request.

## Supplementary Materials

Supplementary Materials Fig. S1: in vivo characterization of ^68^Ga-Nb1159 and preclinical PET imaging analysis of mice model treated with the RBD. (a) Stability of ^68^Ga-Nb1159 in urine and blood in vivo. (b) SUV max value of vital organs in Figure 4(e) KM mouse. (c) Preclinical PET imaging of 18F-FDG injected i.v. into KM mice after subcutaneous injection of the RBD. The white arrow indicates the subcutaneous injection of the RBD. (d) SUV max value of vital organs in Fig. S1c KM mouse. Fig. S2: preclinical PET imaging of KM mice and mice model treated with the RBD. (a) Preclinical PET imaging of ^68^Ga-Nb1159 in KM mouse at 1 h and 2 h. (b) Preclinical PET imaging of ^68^Ga-Nb1159 in KM mouse i.v. at 30 min after intrapulmonary injection of RBD or 0.01 M PBS (left: 300 *μ*g RBD, right: 0.01 M PBS). Fig. S3: preclinical PET imaging of lung and the uptake analysis by gamma-counter for mice model treated with the RBD. (a) Preclinical PET imaging of ^68^Ga-Nb1159 i.v. injected into KM mice at 30 and 60 min after intrapulmonary injection of the RBD (75 *μ*g). (b) The PET imaging of lung in vitro for mice treated with the RBD in Fig. S3a. (c) The PET imaging of lung in vitro for mice treated with 0.01 M PBS. (d) Comparison between the uptakes for lung (%ID/g) in mice treated with RBD and 0.01 M PBS. Fig. S4: preclinical PET imaging and analysis of mice coinjected with Nb16-68 and Nb11-59. (a) Preclinical PET imaging of ^68^Ga-Nb1159 coinjected with nanobodies in KM mouse i.v. after subcutaneous injection of RBD. The white arrow indicates the subcutaneous injection of RBD. (b) Comparison of SUV max in Fig. S4a between mice co-injected with PBS and Nb16-68, respectively. Fig. S5: preclinical PET imaging and analysis of mice model treated with the RBD in other two groups. (a) Preclinical PET imaging of ^68^Ga-Nb1159 i.v. injected into KM mice after subcutaneous injection of the RBD. The white arrow indicates the subcutaneous injection of the RBD. Fig. S6: the T/NT value between the mouse (40 *μ*g RBD) in Figure 4(e) and the mouse (40 *μ*g RBD) coinjected PBS and ^68^Ga-Nb1159 in Figure 5(a). Fig. S7: the amino acid sequence (a), SDS-PAGE data (b), the MALDI-TOF Mass Spectrometric Charts (c) of Nb11-59, and the MALDI-TOF Mass Spectrometric Charts (d) of NOTA-Nb1159.
