# Supplementary material for: Evaluation of SARS-CoV-2-Neutralizing Nanobody Using Virus Receptor Binding Domain-Administered Model Mice
Source: Research (Wash D C). 2022 Jul 22;2022:9864089. doi: 10.34133/2022/9864089 (PMC9343077; doi:10.34133/2022/9864089)
Supplement: Supplementary Materials — Fig. S1: in vivo characterization of 68Ga-Nb1159 and preclinical PET imaging analysis of mice model treated with the RBD. (a) Stability of 68Ga-Nb1159 in urine and blood in vivo. (b) SUV max value of vital organs in Figure 4(e) KM mouse. (c) Preclinical PET imaging of 18F-FDG injected i.v. into KM mice after subcutaneous injection of the RBD. The white arrow indicates the subcutaneous injection of the RBD. (d) SUV max value of vital organs in Fig. S1c KM mouse. Fig. S2: preclinical PET imaging of KM mice and mice model treated with the RBD. (a) Preclinical PET imaging of 68Ga-Nb1159 in KM mouse at 1 h and 2 h. (b) Preclinical PET imaging of 68Ga-Nb1159 in KM mouse i.v. at 30 min after intrapulmonary injection of RBD or 0.01 M PBS (left: 300 μg RBD, right: 0.01 M PBS). Fig. S3: preclinical PET imaging of lung and the uptake analysis by gamma-counter for mice model treated with the RBD. (a) Preclinical PET imaging of 68Ga-Nb1159 i.v. injected into KM mice at 30 and 60 min after intrapulmonary injection of the RBD (75 μg). (b) The PET imaging of lung in vitro for mice treated with the RBD in Fig. S3a. (c) The PET imaging of lung in vitro for mice treated with 0.01 M PBS. (d) Comparison between the uptakes for lung (%ID/g) in mice treated with RBD and 0.01 M PBS. Fig. S4: preclinical PET imaging and analysis of mice coinjected with Nb16-68 and Nb11-59. (a) Preclinical PET imaging of 68Ga-Nb1159 coinjected with nanobodies in KM mouse i.v. after subcutaneous injection of RBD. The white arrow indicates the subcutaneous injection of RBD. (b) Comparison of SUV max in Fig. S4a between mice co-injected with PBS and Nb16-68, respectively. Fig. S5: preclinical PET imaging and analysis of mice model treated with the RBD in other two groups. (a) Preclinical PET imaging of 68Ga-Nb1159 i.v. injected into KM mice after subcutaneous injection of the RBD. The white arrow indicates the subcutaneous injection of the RBD. Fig. S6: the T/NT value between the mouse (40 μg RBD) in Figure 4(e [file 9864089.f1.docx]

**Front Matter**

Title

Evaluation of SARS-CoV-2-neutralizing Nanobody Using Virus Receptor Binding Domain Administered Model Mice

Authors

Song Liu^1,†^, Guanghui Li^2,†^, Lei Ding^3,†^, Jin Ding^1^, Qian Zhang^1^, Dan Li^1^, Xingguo Hou^1^,Xiangxing Kong^1^, Jing Zou ^5,6^, Shiming Zhang ^5,6^, Hongbin Han^4,5,6*^, Yakun Wan^2,*^, Zhi Yang^1,5,7*^, Hua Zhu ^1,5,7*^

Affiliations

^1^ Key Laboratory of Carcinogenesis and Translational Research (Ministry of Education/Beijing), Key Laboratory for Research Evaluation of Radiopharmaceuticals (National Medical Products Administraion), Department of Nuclear Medicine, Peking University Cancer Hospital and Institute, Beijing, 100142, China.

^2^ Shanghai Novamab Biopharmaceuticals Co., Ltd, Shanghai China.

^3^ Key Laboratory of Carcinogenesis and Translational Research (Ministry of Education/Beijing), Department of Anesthesiology, Peking University Cancer Hospital & Institute, Beijing 100142, China.

^4^ Department of Radiology, Peking University Third Hospital, Peking University, Beijing, 100191, China.

**^5^** Institute of Medical Technology, Peking University Health Science Center, Beijing, 100191, China.

^6^ Beijing Key Laboratory of Magnetic Resonance Imaging Devices and Technology, Peking University Third Hospital, Beijing 100191, China.

^7^ Institute of Biomedical Engineering, Peking University Shenzhen Graduate School, Shenzhen, Guangdong 518055, China

Correspondence should be addressed to Hua Zhu; email: [zhuhuaBCH@pku.edu.cn](mailto:zhuhuaBCH@pku.edu.cn) and Zhi Yang; email: [pekyz@163.com](mailto:pekyz@163.com) and Yakun Wan; email: [ykwan@novamab.com](mailto:ykwan@novamab.com) and Hongbin Han; email: hanhongbin@bjmu.edu.cn

Author list:

Song Liu, No. 52 Fu-Cheng Rd., Beijing, 100142, CHINA. Tel.: +86 010 88196495, Fax: +86010 88196393, email: [l](mailto:liusongg1994@163.com)[iusongg1994@163.com](mailto:liusongg1994@163.com).

Guanghui Li, No. 500 FuRong Flower Road, Pudong New District, Shanghai, CHINA. Tel: 86-21-20985259-818, Email: [ghli@novamab.com](mailto:ghli@novamab.com).

Lei Ding, No. 52 Fu-Cheng Rd., Beijing, 100142, CHINA. Tel.: 86-010-88196107, Fax: 86-010-88121122, email: dingleimzk@126.com.

Jin Ding, No. 52 Fu-Cheng Rd., Beijing, 100142, CHINA. Tel.: +86 010 88196495, Fax: +86010 88196393, email: as110_007@163.com.

Qian Zhang, No. 52 Fu-Cheng Rd., Beijing, 100142, CHINA. Tel.: +86 010 88196495, Fax: +86010 88196393, email: 1806708691@qq.com.

Dan Li, No. 52 Fu-Cheng Rd., Beijing, 100142, CHINA. Tel.: +86 010 88196495, Fax: +86010 88196393, email: 17839949055@163.com.

Xingguo Hou, No. 52 Fu-Cheng Rd., Beijing, 100142, CHINA. Tel.: +86 010 88196495, Fax: +86010 88196393, email: 595178260@qq.com.

Xiangxing Kong, No. 52 Fu-Cheng Rd., Beijing, 100142, CHINA. Tel.: +86 010 88196495, Fax: +86010 88196393, email: [13563970213@163.com](mailto:13563970213@163.com).

Jing Zou, No. 38 Xue-Yuan Rd., Beijing, 100191, CHINA. Tel.: +86 010 88196495, Fax: +86010 88196393, email: [18308463391@163.com](mailto:18308463391@163.com).

Shiming Zhang, No. 38 Xue-Yuan Rd., Beijing, 100191, CHINA. Tel.: +86 010 88196495, Fax: +86010 88196393, email: 2111110690@bjmu.edu.cn.

Supplementary Materials

In this article, specific activity (SA) is defined as the radioactivity per unit mass of a labeled nanobody.

In 1 mg ^68^Ga, the number of atoms of N of ^68^Ga is

$$\text{N=}\frac{1\times{10}^{-3}\times6.02\times{10}^{23}}{68}$$

The decay constant λ of ^68^Ga is

$$\lambda=\frac{ln2}{t_{1/2}}=\frac{0.693}{68\times60} s^{-1}$$

In 1 mg ^68^Ga, the specific activity SA of ^68^Ga is

$$SA=\lambda N$$

$$=\frac{0.693\times{10}^{-3}\times6.02\times{10}^{23}}{68\times60\times68} Bq$$

$$=1.50\times{10}^{15} Bq$$

$$=1.50\times{10}^{6}GBq$$

$$=4.05\times{10}^{7}mCi$$

Therefore, the specific activity of ^68^Ga is 4.05 × 10^7^ mCi/mg or 1.50 × 10^6^ GBq/mg.

The total injection mass of ^68^Ga-Nb1159 and Nb11-59 was 4 μg with the activity 0.2 mCi according to calculation. We assumed that one Nb11-59 chelated one ^68^Ga. The radio of ^68^Ga to Nb11-59 (13.36 kDa) in this injection is

$$R=\frac{\frac{0.2\times{10}^{-3}}{68\times4.05\times{10}^{7}}}{\frac{4\times{10}^{-6}}{13.36\times{10}^{3}}}$$

$$=0.02\%$$

The SUVmax is predicated on long term imaging evaluation and statistical comparison of numerous datasets in the field of PET. The value is determined as below:

$$SUVmax=r/(\frac{a^{'}}{w})$$

where r is the maximum radioactivity activity concentration (kBq/ml) measured by the PET scanner within a region of interest (ROI), a′ is the decay-corrected amount of injected radiolabeled tracer (kBq), and w is the weight of the body (g), which is used a surrogate for a distribution volume of tracer. This provides a metric for comparison across the bodies at different times.


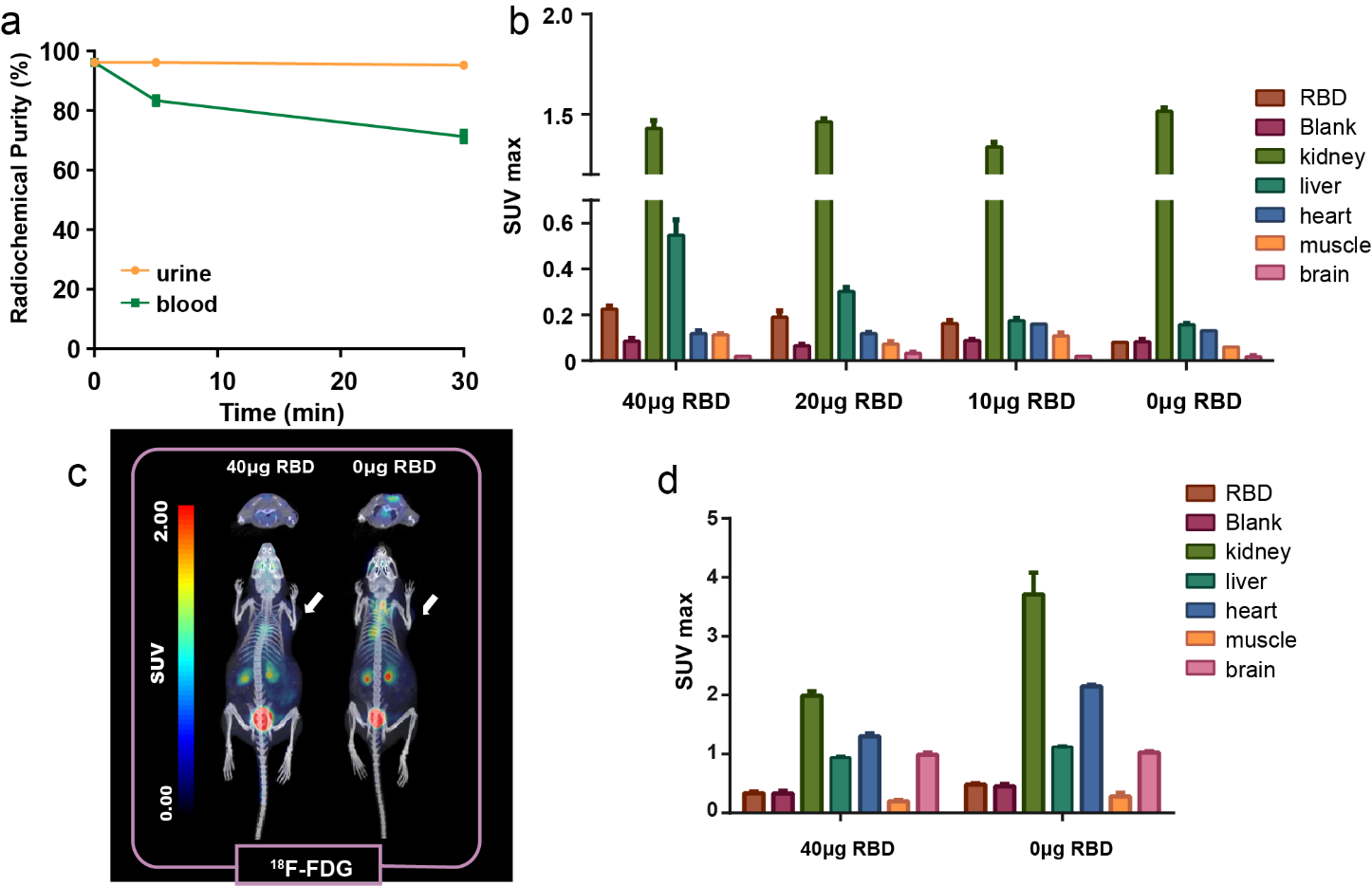


**Fig. S1**. ***In vivo* characterization of ^68^Ga-Nb1159 and preclinical PET imaging analysis of mice model treated with the RBD.** (a) Stability of ^68^Ga-Nb1159 in urine and blood *in vivo*. (b) SUV max value of vital organs in Fig. 4e KM mouse. (c) Preclinical PET imaging of ^18^F-FDG injected i.v. into KM mice after subcutaneous injection of the RBD. The white arrow indicates the subcutaneous injection of the RBD. (d) SUV max value of vital organs in Fig. S1c KM mouse.


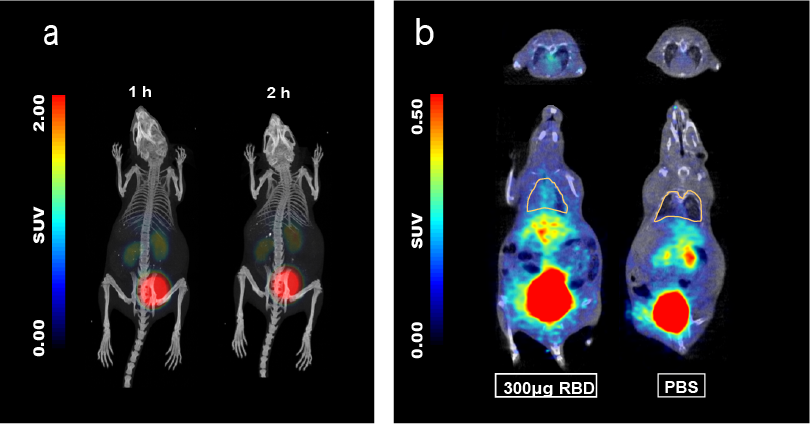


**Fig. S2**. **Preclinical PET imaging of KM mice and mice model treated with the RBD.** (a) Preclinical PET imaging of ^68^Ga-Nb1159 in KM mouse at 1h and 2h. (b) Preclinical PET imaging of ^68^Ga-Nb1159 in KM mouse i.v. at 30 min after intrapulmonary injection of RBD or 0.01 M PBS (left: 300 μg RBD, right: 0.01 M PBS).


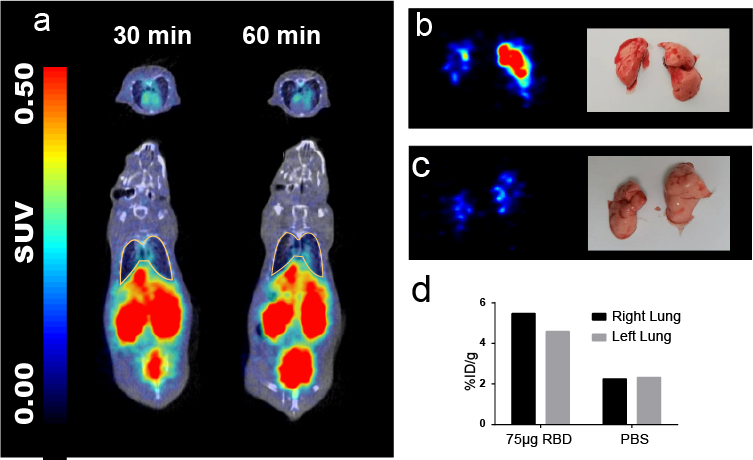


**Fig. S3. Preclinical PET imaging of lung and the uptake analysis by gamma-counter for mice model treated with the RBD.** (a) Preclinical PET imaging of ^68^Ga-Nb1159 i.v. injected into KM mice at 30 and 60 min after intrapulmonary injection of the RBD (75 μg). (b) The PET imaging of lung *in vitro* for mice treated with the RBD in Fig S3a. (c) The PET imaging of lung *in vitro* for mice treated with 0.01 M PBS. (d) Comparison between the uptake for lung (%ID/g) in mice treated with RBD and 0.01 M PBS.


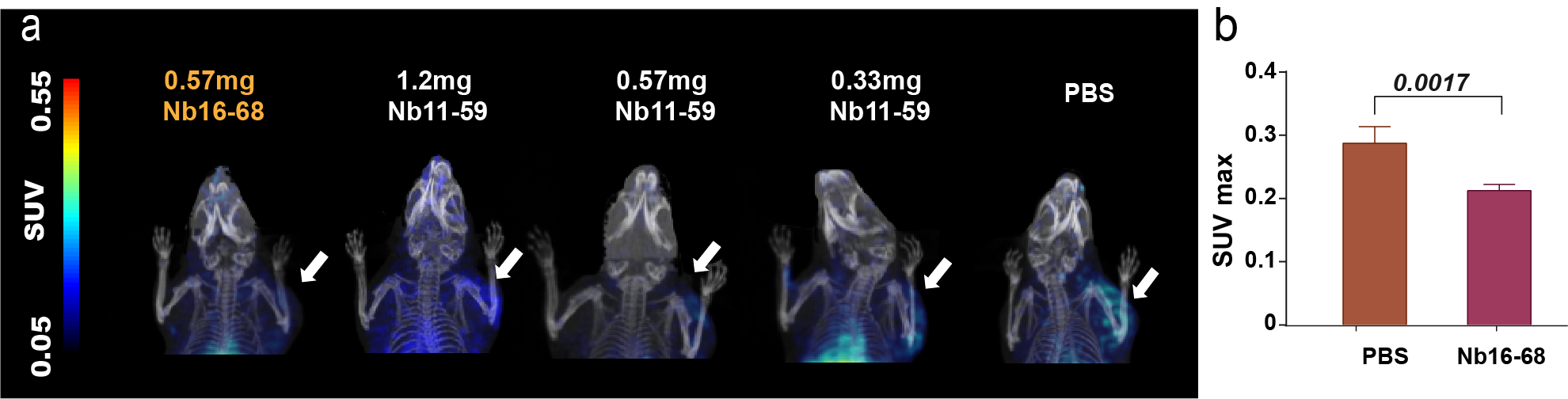


**Fig. S4**. **Preclinical PET imaging and analysis of mice co-injected with Nb16-68 and** **Nb****11-59.** (a) Preclinical PET imaging of ^68^Ga-Nb1159 co-injected with nanobodies in KM mouse i.v. after subcutaneous injection of RBD. The white arrow indicates the subcutaneous injection of RBD. (b) Comparison of SUV max in Fig. S4a between mice co-injected with PBS and Nb16-68, respectively.


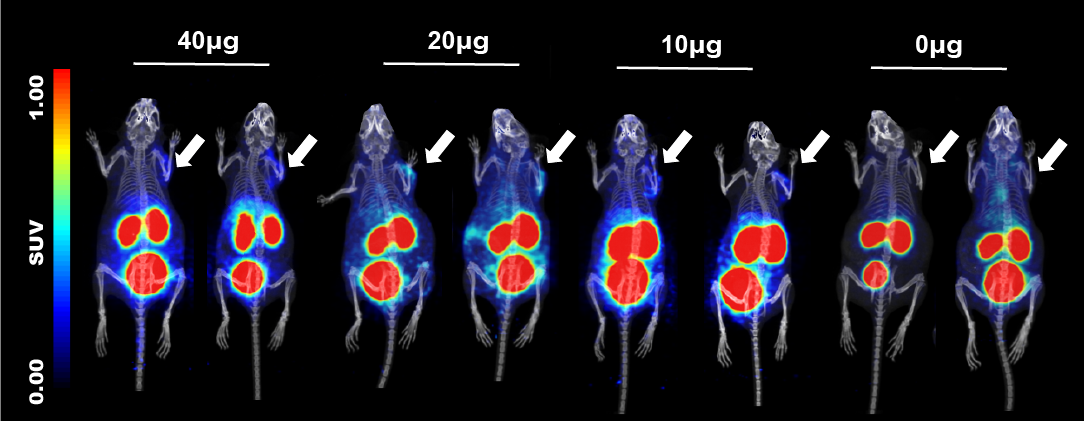


**Fig. S5. Preclinical PET imaging and analysis of mice model treated with the RBD in other two groups.** (a) Preclinical PET imaging of ^68^Ga-Nb1159 i.v. injected into KM mice after subcutaneous injection of the RBD. The white arrow indicates the subcutaneous injection of the RBD.


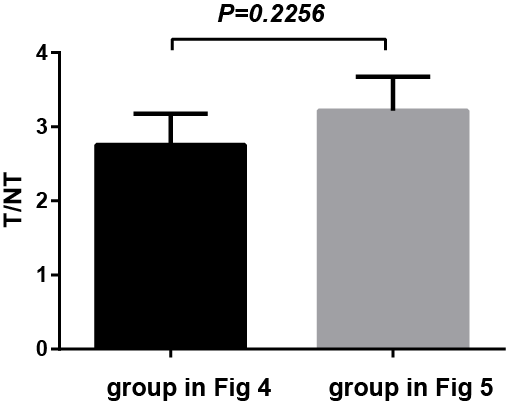


**Fig. S6. The T/NT value between the mouse (40 μg RBD) in Fig.4e and the mouse (40 μg RBD) co-injected PBS and ^68^Ga-Nb1159 in Fig.5a.**


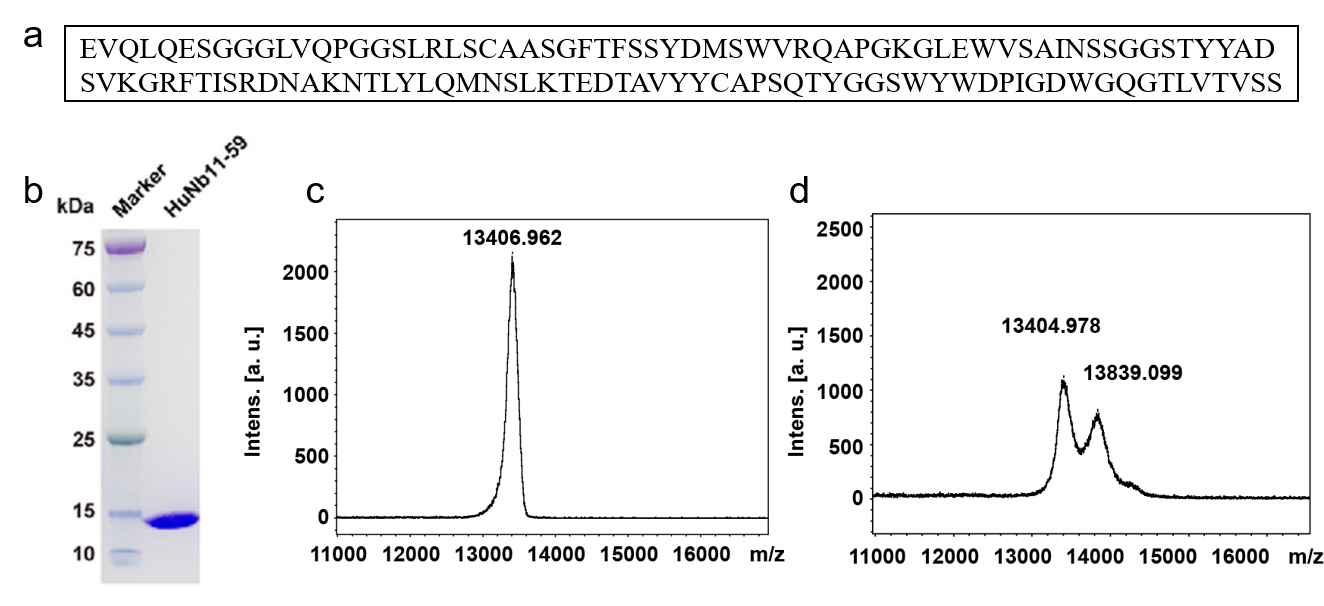


**Fig. S7. The amino acid sequence (a), SDS-PAGE data (b), the MALDI-TOF Mass Spectrometric Charts (c) of Nb11-59, and the MALDI-TOF Mass Spectrometric Charts (d) of NOTA-Nb1159.**
